# Organogermanium: Potential beneficial effects on the cardiovascular system

**DOI:** 10.14814/phy2.70234

**Published:** 2025-02-04

**Authors:** Kunihiko Aizawa, Takashi Nakamura, Yasuhiro Shimada, Tomoya Takeda, Junya Azumi, Angela C. Shore

**Affiliations:** ^1^ Department of Clinical and Biomedical Sciences University of Exeter Medical School Exeter UK; ^2^ NIHR Exeter Clinical Research Facility Exeter UK; ^3^ Asai Germanium Research Institute Co. Ltd. Hakodate Hokkaido Japan

**Keywords:** artery, blood pressure, diabetes, erythrocyte, inflammation, oxidative stress

## Abstract

Organogermanium, especially poly‐*trans*‐[(2‐carboxyethyl)germasesquioxane] (Ge‐132), has been known to enhance immune‐modulatory activities. However, the in vivo and in vitro evidence accumulated over the last 20 years reveals that Ge‐132 has unique but underappreciated multi‐functional properties that have a potential positive effect for the cardiovascular system. A hydrolysate of Ge‐132, monomeric 3‐(trihydroxygermyl)propanoic acid, forms a complex with a vicinal diol structure (i.e., having two adjacent hydroxyl groups such as the *cis*‐diol and catechol groups) that exists in ribose (e.g., adenosine triphosphate), catecholamine (e.g., adrenaline), and saccharide (e.g., glucose). Additionally, Ge‐132 enhances macrophage phagocytosis and the heme catabolic pathway by upregulating key enzymes that are responsible for producing cytoprotective molecules such as biliverdin and bilirubin during the process. These multi‐functional properties exert pleiotropic physiological effects after an oral intake of Ge‐132 such as anti‐oxidation, anti‐inflammation, anti‐hypertensive, anti‐glycation, and erythrocyte lifecycle enhancement, all of which appear to assist the cardiovascular system. Of those effects, the effects on the lifecycle of erythrocyte may have an important implication for maintaining optimal vascular function, augmenting the availability of oxygen by enhancing the elimination of senescent, and damaged erythrocytes as well as promoting erythropoiesis. Human studies are warranted to determine whether these beneficial effects observed in previous studies are translated into humans.

## 1 INTRODUCTION

Germanium is a versatile natural trace element with diverse applications. Germanium compounds once gained prominence in the electronics industry, but it has become apparent that they possess a variety of biological and potentially medicinal activities. The first description of bioactivities by germanium was the demonstration that the inorganic germanium compound germanium dioxide (GeO_2_) potentiated erythropoiesis in 1922 (Hammett et al., 1922). But the toxicity of GeO_2_ severely limits its use in humans. Soon after Mironov et al. (1967) successful synthesis of an organogermanium compound, a water‐soluble organogermanium compound was sought after because one was predicted to have a potential pharmaceutical use. Asai and his colleagues synthesized the desired compound and confirmed its crystal structure, named Ge‐132 (poly‐*trans*‐[(2‐carboxyethyl)germasesquioxane]) (Tsutsui et al., 1976). It has since been clinically examined for its immunological effects, most well‐studied of which is the immune‐modulating activity of Ge‐132 which shows the activation of macrophages and natural killer cells leading to an increased plasma interferon γ level following an oral intake of Ge‐132 (Aso et al., 1985).

Unfortunately, there were fatal accidents related to the oral intake of high‐dose *inorganic* germanium compounds (GeO_2_ and germanium citrate/lactate) due to unscrupulous business practices in the 1980s including the marketing of Ge‐132 supplements contaminated with GeO_2_ and germanium citrate/lactate. These practices disheartened the public and a decline of scientific/medical interests in exploring the medicinal use of organogermanium compounds was seen in the ensuing years (Kaplan et al., 2004). However, this unfortunate trend did not deter researchers' enthusiasm to explore effects of Ge‐132, and because Ge‐132 had been used for food supplements (Nakamura et al., 2023), researchers started to explore the effects of Ge‐132 as a food supplement.

Together with its previously observed immuno‐stimulatory activities, multi‐functional properties of Ge‐132 have since been discovered in animal models which are uncommon in other food supplements. These unique but underappreciated properties of Ge‐132 may have potential benefits for the cardiovascular system including arterial stiffness, atherosclerosis, and blood circulation. However, evidence to support this contention in humans is currently absent due to a lack of research studies conducted in humans. Therefore, the aim of this focused review is to raise awareness of the multiple effects that the food supplement Ge‐132 has in in vivo models and in vitro with the hope of stimulating researchers' interest in this compound. Here, we mainly focus on the research studies conducted with a specific Ge‐132 compound (Asaigermanium®) because it has repeatedly been proven through various safety tests as a safe compound, the topic of which will be discussed in the next section.

## 2 PHARMACOKINETICS AND SAFETY PROFILE OF ORGANOGERMANIUM

Before introducing the properties of Ge‐132, we touch upon the characteristics, pharmacokinetics and safety of Ge‐132 (Nakamura et al., 2023). Ge‐132 is colorless crystals/crystalline powder, which is odorless and tastes slightly sour. it is decomposed above 270°C. Ge‐132 is soluble in water (1.09% at 20°C) and is extremely soluble in an alkaline environment where 10% or more of Ge‐132 is dissolved at pH 7.4. It is insoluble or very poorly soluble in most organic solvents. 3‐(trihydroxygermyl)propanoic acid (THGP) is not metabolized in the liver nor in the intestine by gut bacteria after the oral intake. In rats, approximately 20% of orally administered Ge‐132 is absorbed in the gastrointestinal tract. Because Ge‐132 is extremely soluble in the alkaline environment, it is mostly hydrolyzed in the duodenum into monomeric THGP. THGP is absorbed via intestinal epithelial cells, and plasma THGP concentration peaks ~3 h after the oral intake. The absorbed THGP is then excreted in the urine. Within 24–48 h, THGP is almost completely excreted without any accumulation in the body.

Median lethal dose (LD_50_) of Ge‐132 when orally ingested is extremely high; >14,489 mg/kg for rat and >8500 mg/kg for dog, which when converted to a 60‐kg human are 869 and 510 g, respectively (more than 1000–1500 times the suggested daily intake) (Nakamura et al., 2023). The safety of Ge‐132 has been confirmed in rats through various safety tests and by Good Laboratory Practice (GLP) compliance toxicity tests (Doi et al., 2017; Iwadate et al., 2018). In addition, Ge‐132 (Asaigermanium) has recently been certified and registered with a third‐party certification programme (the Japan Health and Nutrition Food Association) for the safety of health foods by the Japanese version of self‐affirmed Generally Recognized As Safe (GRAS) designation. Therefore, the safety of Ge‐132 as a food supplement is firmly established.

There were multiple fatal incidents during the 1980s that were related to high‐dose oral intake of *inorganic* germanium GeO_2_ and germanium citrate/lactate (Sanai et al., 1990), which have still cast a shadow over organogermanium products in general (Krapf et al., 1992). Nephrotoxicity of GeO_2_ resulting in acute and chronic renal failure was the main cause of fatalities for both inorganic germanium compounds, but they were also reported to cause other issues such as liver dysfunction, myopathy and neuropathy (Higuchi et al., 1991; Sanai et al., 1990). As for germanium citrate/lactate, these inorganic germanium compounds were erroneously labeled and sold as “organogermanium” which is not the case as they do not contain any Ge‐C bonds that are characteristics of organogermanium. Furthermore, it has been reported that GeO_2_ is generated in the process of hydrolysis of germanium citrate/lactate in water (Sato et al., 2012), which explains the similarities of renal and hepatic toxicity caused by germanium citrate/lactate and GeO_2_ (Krapf et al., 1992). Had these inorganic germanium compounds been put through toxicity tests, these unfortunate fatalities associated with GeO_2_ and germanium citrate/lactate could have probably been avoided. Therefore, it cannot be emphasized enough that, prior to putting their products on the market, manufacturers of organogermanium products should recognize the importance of safety and conduct extensive toxicity tests, the results of which should also be transparently communicated with end users.

## 3 MULTI‐FUNCTIONAL PROPERTIES OF ORGANOGERMANIUM

In this section, three main properties of Ge‐132 will be focused on that may have beneficial effects on the cardiovascular system in humans.

### 3.1 Interaction with bioactive molecules

**FIGURE 1:**
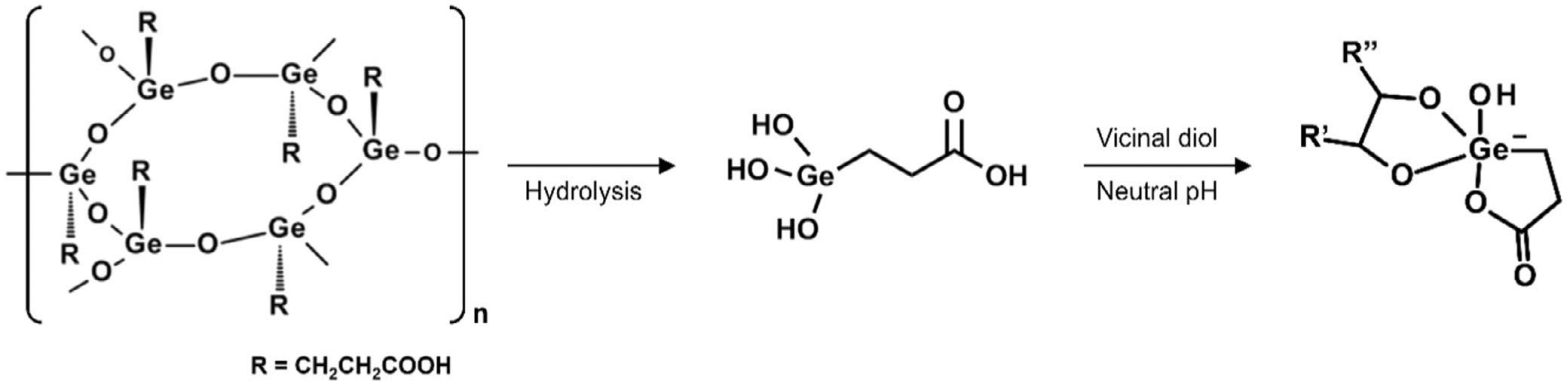
Schematic diagram of the complex formation reaction between the hydrolysate of Ge‐132, THGP, and vicinal diol compounds. THGP reacts with vicinal diol compounds through a dehydration reaction via the Ge‐OH group in the molecule, forming stable complexes in a neutral pH aqueous solution. THGP, 3‐(trihydroxygermyl)propanoic acid.

After an oral intake of Ge‐132, THGP is formed in the gastrointestinal tract following the hydrolysis reaction. It is thus THGP that exerts the biological activities of Ge‐132 in the body in animals. THGP forms a loose and reversible complex with many molecules that perform an essential function in humans. Specifically, THGP forms a complex with a vicinal diol structure (i.e., having two adjacent hydroxyl groups such as the *cis*‐diol and catechol groups) that exists in ribose (e.g., adenosine and adenosine triphosphate [ATP]), catecholamine (e.g., adrenaline and dopamine) and saccharide (e.g., glucose and fructose) (Nakamura et al., 2015). We have previously demonstrated that THGP forms a complex with *cis*‐diol groups but not with *trans*‐diol groups (Shimada et al., 2021), and that THGP interacts with l‐3,4‐dihydroxyphenylalanine (l‐DOPA; which contains a catechol group) but not with tyrosine (a phenol‐derivative of l‐DOPA without a catechol group) (Azumi et al., 2019). The complex formation observed above were reversible and showed a dependency on substrate concentrations. Figure 1 shows an example of complex formation between THGP and a vicinal diol structure. The complex formation between THGP and bioactive molecules diminishes binding of the molecules to respective receptors, suppressing signal transduction downstream (Nakamura et al., 2015).

### 3.2 Enhancement of macrophage phagocytosis

A recent study has demonstrated that M0 macrophage under a long‐term co‐culture with THGP was found to differentiate into M1‐type macrophage when activated through NF‐κB (Azumi et al., 2023). A long‐term co‐culture of RAW 264.7 mouse macrophage‐like cells with THGP also resulted in the increased phagocytic capability toward B16 4A5 melanoma cells in vitro (Azumi et al., 2023). These findings help explain the underlying molecular mechanisms of anti‐cancer effects previously observed in vivo and ex vivo with Ge‐132 (Nakamura et al., 2023). Furthermore, the enhanced phagocytosis of senescent erythrocytes by macrophages has been observed following consumption of a diet containing Ge‐132 in mice (Takeda et al., 2024). Intriguingly, hematocrit levels in the Ge‐132 diet‐fed mice were similar to those in the chow diet‐fed mice despite the increased erythrocyte phagocytosis, suggesting the possibility that Ge‐132 may also promote erythropoiesis, similar to GeO_2_, to maintain erythrocytes homeostasis.

### 3.3 Enhancement of the heme catabolic pathway

Upon phagocytosis by macrophages, erythrocytes release heme, which is metabolized to biliverdin by heme oxygenase 1 (HO‐1), carbon monoxide (CO) which has vasodilatory as well as anti‐inflammatory effects, and ferrous iron. Biliverdin reductase (BLVR) then reduces biliverdin to antioxidant bilirubin. Following the glucuronidation by uridine diphosphate glucuronyltransferase 1A1 (UGT1A1), bilirubin is excreted through the bile ducts to the duodenum and subsequently transported to the large intestine where it is deconjugated and further reduced by gut bacteria to urobilinogen, another antioxidant with higher radical scavenging capacity (Nakamura et al., 2006). The oral intake of Ge‐132 upregulates the activity of these key enzymes (HO‐1, UGT1A1, and very likely BLVR) in the heme catabolic pathway in mice (Takeda et al., 2024), precipitating the production of vasodilatory/anti‐inflammatory (CO), and antioxidant (bilirubin and urobilinogen) substances that individually or collectively contribute to the cytoprotective action of this pathway.

## 4 BENEFICIAL PHYSIOLOGICAL EFFECTS OF ORGANOGERMANIUM SUPPLEMENTATION ON CARDIOVASCULAR HEALTH

The unique properties of Ge‐132 illustrated above are the cornerstone of diverse physiological actions that Ge‐132 exerts as a food supplement.

### 4.1 Antioxidative effect

Ge‐132 itself does not have a direct radical scavenging capability in contrast with typical antioxidants such as ascorbic acid (Nakamura et al., 2010; Takeda et al., 2019). Bilirubin itself is a vital antioxidant, but as discussed above it is converted by gut bacteria to a potent antioxidant urobilinogen in the large intestine (Hall et al., 2024; Nakamura et al., 2006). Radical scavenging activity of fecal stercobilinogen (a structural isomer of urobilinogen) in Ge‐132 fed mice was found to be ~3000 folds higher than that of vitamin C when calculated at a daily recommended dose (90 mg of vitamin C for male adults) (Takeda et al., 2024). These antioxidants may counteract the impact of food‐derived radical species in the large intestine and help keep the intestinal environment from the damage associated with oxidative stress. Intriguingly, there is some in vitro evidence of HO‐1 facilitating antioxidative defense independently of its enzymatic function (i.e., independently of the heme catabolic pathway); an induction of nuclear translocation of truncated HO‐1 protein under oxidative stress that activates transcription factors and may play a pivotal role in cytoprotection against oxidative stress (Biswas et al., 2014; Lin et al., 2007). It is, however, currently unknown whether Ge‐132 also exerts an antioxidative effect via this nonenzymatic HO‐1 route.

### 4.2 Anti‐inflammatory effect

Although an anti‐inflammatory effect of Ge‐132 has been documented since its early days of medicinal development phase for cancer treatment, underlying anti‐inflammatory mechanisms of Ge‐132 intake was hitherto unclear. Azumi and colleagues have recently identified an in vitro molecular mechanism of anti‐inflammatory action by Ge‐132 demonstrating that THGP forms the complex with ATP to suppress ATP's binding to the P2X7 receptor as a ligand (termed the Damage/Danger Associated Molecular Patterns: DAMPs), binding of which would activate NLRP3 inflammasome (Azumi et al., 2022). The THGP‐ATP complex formation thus suppresses the activation of the NLRP3 inflammasome cascade, reducing secretion of inflammatory cytokine IL‐1β that contributes to pyroptosis (Takahashi, 2022). This unique mechanism of anti‐inflammatory action is in contrast with those anti‐inflammasome inhibitors under development that antagonize ATP receptors or inhibit NLRP3 activity (Shao et al., 2015). Of note, THGP has also been shown to lower IL‐1β secretion independently of THGP‐ATP complex formation, though to a lesser extent than the ATP‐dependent pathway, suppressing the priming of NLRP3 inflammasome (Azumi et al., 2022). The molecular mechanism of this ATP‐independent suppressing effect by Ge‐132 is currently unclear but might include the inhibition of NF‐κB and MAPK activation by Ge‐132 (Wang et al., 2020), and/or the anti‐inflammatory effect of CO.

### 4.3 Anti‐hypertensive effect

Because catecholamines contain the catechol group, they form complexes with THGP with higher affinity than that for nucleosides/nucleotides (76% vs. 22%, respectively) (Nakamura et al., 2023). Catecholamines normally mediate their constrictor effect by binding to α_1_‐adrenergic receptors located mainly on the vascular smooth muscle cell (Motiejunaite et al., 2021). Pharmacological α_1_‐adrenoceptor antagonists induce vasodilatation and lower blood pressure (BP) by blocking the binding of catecholamines to α_1_‐adrenergic receptors (Motiejunaite et al., 2021). In contrast, complex formation between THGP and adrenaline/noradrenaline is considered to suppress their binding to α_1_‐adrenergic receptors as well as downstream signaling cascades that cause vasoconstriction, leading to vasodilatation and BP lowering (Nakamura et al., 2015). The BP lowering effect of Ge‐132 was observed in spontaneously hypertensive rats after a month of oral Ge‐132 ingestion (Sato & Ishikawa, 1973) and in Sprague–Dawley rats following an intraperitoneal injection (Ho et al., 1990). The latter study showed a dose‐dependent effect of BP and heart rate lowering by Ge‐132 (Ho et al., 1990).

It is possible that the complex formation between THGP and adrenaline could inhibit adrenaline's binding to and activation of β_2_‐adrenergic receptors in coronary arteries that would lead to vasoconstriction. Indeed, there is a report showing a reduced peristalsis following an administration of organogermanium to ileum extracted from rabbits (Tomizawa et al., 1978). However, because of the fact that the complex formation depends on the concentration of THGP and it is diluted in the blood, it is likely that the inhibiting effect of THGP/adrenaline complex on β_2_‐adrenergic receptors would be weak. The observation that a 1‐month oral Ge‐132 supplementation in healthy adults did not alter BP levels partly supports the concept (unpublished observation). Similarly, an intra‐venous infusion of Ge‐132 did not alter BP levels in healthy rabbits (Tomizawa et al., 1978). Of course, future investigation on this issue will be warranted.

Regarding an effect of THGP and adrenaline complex on β_1_‐adrenergic receptors, it is also plausible that the complex formation between THGP and adrenaline could inhibit adrenaline's binding to, and activation of, β_1_‐adrenergic receptors in the heart, potentially influencing inotropy and chronotropy. Nevertheless, there is a paucity of research directly investigated in this point and this should be clarified in future.

Although not strictly related to anti‐hypertensive effects but to vascular function, it is conceivable that the THGP/ATP complex could also act on the vasculature but might have different effects depending on the vascular bed, that is, promoting vasodilation for some vascular beds or vasoconstriction for others.

### 4.4 Anti‐glycation effect

Ge‐132 itself does not have a glucose‐lowering ability and thus does not directly alter plasma glucose concentrations. Increased formation of advanced glycation end products (AGEs) due to hyperglycemia is the hallmark of diabetic complications, resulting from enzymatic and nonenzymatic interactions between glucose and proteins/lipids that can induce a cross‐linking of collagen in the vascular wall increasing large arterial stiffness (Aizawa et al., 2024) as well as contribute to microvascular complications of diabetes in humans (Chilelli et al., 2013). Based on the result obtained using a nuclear magnetic resonance spectroscopy, Ge‐132 appears to exert its anti‐glycation effect by the complex formation between THGP and glucose/fructose (Masaki et al., 2024; Shimada et al., 2015). Furthermore, THGP is also reported to form a complex with Amadori compounds, suggesting that Ge‐132 might modulate downstream signaling cascades associated with the formation of AGEs (Osawa et al., 1994). Complex formation between THGP and excess glucose or Amadori compounds thus reduces the substrate available for AGEs synthesis, indicating a slowing of AGEs production as well as reduction of AGEs‐derived oxidative stress and inflammatory response (Nakamura et al., 1999). In a rat model, the prevention of AGEs formation by Ge‐132 is considered to be the underlying mechanism of delayed galactose‐induced cataract progression (Unakar et al., 1995). Additionally, Ge‐132 has been shown to lower plasma glycated albumin concentrations in streptozotocin‐induced diabetic rats (Shimojo et al., 1993). Furthermore, reduced AGEs formation by Ge‐132 in a hyperglycaemic condition could prevent erythrocytes from attachment and engulfment by the endothelium through the receptor for AGEs (Catan et al., 2019; Turpin et al., 2020), potentially reducing a predisposition toward the development of atherosclerosis.

On a side note, the complex formation between THGP and glucose/fructose, which is a reversible reaction, requires both THGP and substrates in a relatively high concentration. Because this reaction does not directly reduce plasma glucose concentrations, there is no risk associated with hypoglycemia when organogermanium is administered to normo‐glycaemic individuals.

### 4.5 Effects on lifecycle of erythrocytes

**FIGURE 2:**
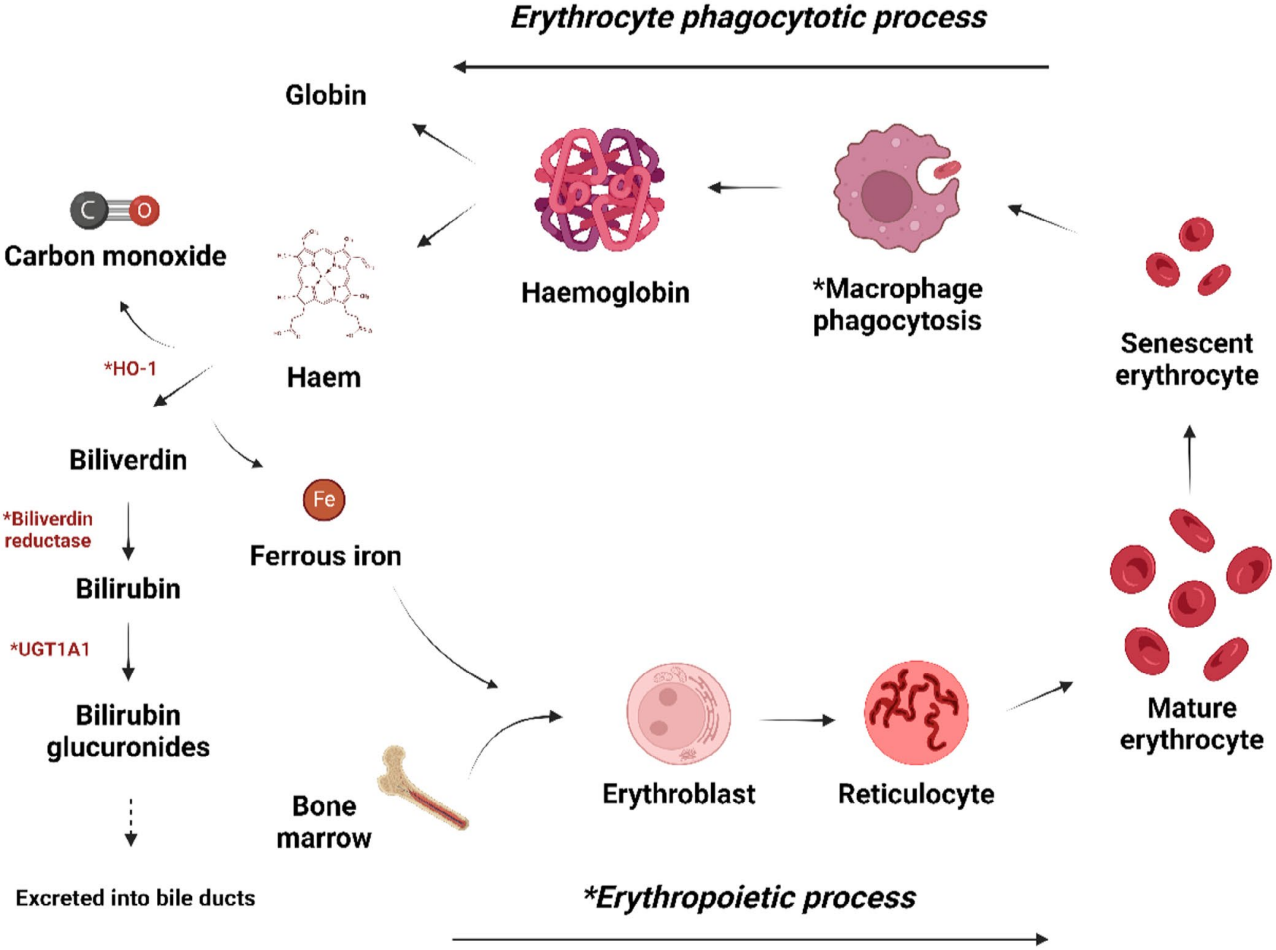
Schematic representation of erythrocyte lifecycle and heme catabolic pathway. See the main text for details. “*” Denotes a site where Ge‐132 may act in the pathway. HO‐1, heme oxygenase 1; UGT1A1, uridine diphosphate glucuronyltransferase 1A1. The figure was created with BioRender.com.

The average human erythrocyte lifespan is around 120 days, during which oxygen molecules repeatedly bind to hemoglobin in the lungs and are released to cells in the body. Due to their abundance in the circulation as well as being under constant risk of endogenous/exogenous oxidative stress, the integrity of the erythrocyte membrane becomes damaged in the course of their lifetime. The damage as they age coincides with the loss of the erythrocyte's deformability, impairing tissue perfusion and oxygenation (Tan et al., 2020). Therefore erythrocytes are considered to play a critical role in the pathophysiology of the cardiovascular system. As described earlier and shown in Figure 2, Ge‐132 promotes the phagocytosis of senescent/damaged erythrocytes by macrophages in the reticuloendothelial system that removes dysfunctional erythrocytes from the circulation (Video S1) (Takeda et al., 2024). Furthermore, the enhanced erythrocyte phagocytosis by macrophages is counter‐balanced by the increased erythrocyte production associated with Ge‐132 intake (Takeda et al., 2024). It remains to be seen whether the underlying mechanism of erythropoiesis with Ge‐132 is an increased production of erythropoietin or erythroblast proliferation by macrophages (Rhodes et al., 2008). Whatever the mechanism may be, replacing senescent/damaged erythrocytes with young/fresh ones should improve tissue perfusion and oxygenation. Since many molecules produced, as well as enzymes that act during the heme catabolic pathway, have antioxidative and anti‐inflammatory effects, the enhancement of erythrocyte lifecycle could be expected to synergistically provide beneficial effects on the microvascular function of vital organs such as the kidneys and brain. In terms of glycated erythrocytes, the attachment and engulfment of glycated erythrocytes by the endothelium is associated with an increased phosphatidylserine exposure which is an “eat me” signal on the surface of glycated erythrocytes (Turpin et al., 2020). Recently, Ge‐132 has been demonstrated to indirectly suppress CD47 (a “don't eat me” signal) expression on the surface of B16 4A5 melanoma cells in parallel with the suppression of signal‐regulatory protein alpha (SIRP‐α, another “don't eat me” signal) expression on the surface of macrophages which binds to CD47 to inhibit phagocytosis (Azumi et al., 2023). Given that Ge‐132 promotes phagocytosis through HO‐1 with indirectly suppressed CD47/SIRP‐α expressions and that glycated erythrocytes highly express phosphatidylserine on the surface favoring phagocytosis, the erythrocyte lifecycle enhancement with Ge‐132 could have potential implications for improving vascular performance.

## 5 PERSPECTIVES

Ge‐132 exerts a diverse array of physiological effects not by directly acting as an agonist/antagonist to promote/inhibit downstream signaling pathways, but by interacting with molecules vital for our survival and upregulating innate immunity to promote phagocytosis and the heme catabolic pathway. These unique properties of Ge‐132 form a foundation for its pleiotropic effects, which is noteworthy for a food supplement. Importantly, Ge‐132's safety profile may stem from this ability of THGP to form complexes with substrates rather than to target or affect proteins (Shimada et al., 2018).

One unique feature of Ge‐132 is the enhancement of erythrocyte lifecycle in animals and humans. In the early days of the Ge‐132 research where oxygen insufficiency was believed to be a potential instigator of cancer, the working hypothesis then was that Ge‐132 would have augmented the availability of oxygen by Ge‐132's working in place of oxygen to prevent the onset of cancer (Nakamura et al., 2023). It is now apparent that Ge‐132 does not directly replace oxygen nor does it bind to erythrocytes to improve oxygenation; rather, it appears to augment the availability of oxygen by enhancing the elimination of senescent/damaged erythrocytes as well as promoting erythropoiesis, that is, erythrocyte renewal, not to mention the enhanced production of molecules and enzymes that exerts cytoprotection throughout this erythrocyte renewal process (Takeda et al., 2024). Erythrocyte's lifecycle enhancement has not attracted much attention, probably due to a perception that the erythrocyte is a just simple oxygen carrier, and also the lack of compounds that could act both as an enhancer for erythrocytes' removal and a promotor for erythropoiesis. Given that the erythrocyte is the most abundant cell in the human body traveling throughout the circulatory system and playing a critical role in the pathophysiology of cardiovascular disease, the erythrocyte lifecycle enhancement by Ge‐132 could have an important implication for the cardiovascular function. This would apply not only to at‐risk individuals (e.g., hypertension, type 2 diabetes, cardiovascular disease, etc) but also to those who pursue healthy aging through maintaining optimal vascular function. On a similar note, it can be speculative that erythrocyte lifecycle enhancement by Ge‐132 may favorably influence exercise performance and recovery from exercise bouts via augmented oxygen availability and enhanced erythrocyte's deformability to improve microvascular perfusion (Ebrahimi & Bagchi, 2022).

Currently, Ge‐132 (Asaigermanium) has been utilized as a material for food supplements and cosmetics in Japan and several Asian countries. Furthermore, food supplements labeled as Ge‐132 are on the market in the United States, but the safety of those products are uncertain. As we mentioned above, Ge‐132 (Asaigermanium) is the only organogermanium product so far, in which safety has been confirmed and been externally certified as a food supplement. Further research on the safety as well as biological effects of Ge‐132 is thus warranted to address continued concerns over Ge‐132. Moreover, because the majority of human studies to date have been conducted in Asians (especially in Japanese), it is necessary to determine whether ethnicity influences physiological effects of Ge‐132 that may help determine an optimal dose of Ge‐132 as a supplement. Although Ge‐132 is not an essential nutrient, incorporating Ge‐132 in our daily lives as a food supplement appears promising to assist cardiovascular system health given the beneficial physiological effects described in this review.

Although these fall outside the scope of this review, there are other beneficial physiological effects of Ge‐132 that have recently been identified; these include the protection against oxidative stress‐induced cell death in dermal fibroblasts of human normal skin (Takeda et al., 2019), and the suppression of sulfide‐dependent pain through the complexation between THGP and sulfide (Sekiguchi et al., 2023). The latter is another example of molecular interaction between THGP and bioactive molecules that results in a suppression of downstream signal transduction. There are likely to be other bioactive molecules that could interact with THGP and thus induce (currently unrealised) physiological effects. The use of the liquid chromatography–tandem mass spectrometry technique that enables quantification of minute amount of THGP could help evaluate molecules potentially interacting with THGP and their physiological effects (Yamaguchi et al., 2015).

## FUNDING INFORMATION

No funding information provided.

## CONFLICT OF INTEREST STATEMENT

TN received remuneration as an officer from Asai Germanium Research Institute Co., Ltd. YS, TT, and JA are employees at Asai Germanium Research Institute Co., Ltd. Other authors report no conflict of interest.

## Supporting information


Video S1.
