# Supplementary material for: Organogermanium: Potential beneficial effects on the cardiovascular system
Source: Physiol Rep. 2025 Feb 4;13(3):e70234. doi: 10.14814/phy2.70234 (PMC11794241; doi:10.14814/phy2.70234)
Supplement: Supplementary file 1 — Video S1. [file PHY2-13-e70234-s001.zip › Supplemental video legend.docx]

**Supplemental video** **S1:** Phagocytosis of senescent erythrocytes by macrophages treated with THGP (saved in ***figshare***, <https://figshare.com/s/dec3ae3bbc7d6e50a1bd>).
